# Substantial Narrowing on the Width of “Concentration Window” of Hydrothermal ZnO Nanowires via Ammonia Addition

**DOI:** 10.1038/s41598-019-50641-y

**Published:** 2019-10-02

**Authors:** Daiki Sakai, Kazuki Nagashima, Hideto Yoshida, Masaki Kanai, Yong He, Guozhu Zhang, Xixi Zhao, Tsunaki Takahashi, Takao Yasui, Takuro Hosomi, Yuki Uchida, Seiji Takeda, Yoshinobu Baba, Takeshi Yanagida

**Affiliations:** 1 Institute for Materials Chemistry and Engineering, Kyushu University, 6-1 Kasuga-koen, Kasuga Fukuoka, 816-8580 Japan; 2 Institute of Scientific and Industrial Research, Osaka University, 8-1 Mihogaoka, Ibaraki Osaka, 567-0047 Japan; 3 Japan Science and Technology Agency (JST), PRESTO, 4-1-8 Honcho, Kawaguchi Saitama, 332-0012 Japan; 4 Key Laboratory of Optoelectronic Technology and Systems of the Education Ministry of China, Chongqing University, Chongqing, 400044 P.R. China; 5 Department of Biomolecular Engineering, Graduate School of Engineering, Nagoya University, Furo-cho, Chikusa-ku, Nagoya 464-8603 Japan

**Keywords:** Nanowires, Nanowires

## Abstract

A crystal growth of hydrothermal ZnO nanowires essentially requires a concentration control within so-called “concentration window”, where the anisotropic crystal growth of ZnO nanowires preferentially occurs. Although understanding what exactly determines the width of “concentration window” is important to tailor the anisotropic crystal growth process, the fundamental knowledge as to “concentration window” is still scarce. Here we report the effect of ammonia addition on the width of “concentration window” using conventional hydrothermal ZnO nanowire growth. We found that the ammonia addition substantially narrows the width of “concentration window”. Within the narrow range of zinc complex concentration, we found a significant increase of growth rate (up to 2000 nm/h) of ZnO nanowires. The narrowed “concentration window” and the resultant increased growth rate by the ammonia addition can be understood in terms of synchronized effects of both (1) a reduction of zinc hydroxide complex (precursor) concentration and (2) a fast rate limiting process of ligand exchange between different zinc complexes. Thus, the present knowldege as to “concentration window” will accelerate further tailoring an anisotropic crystal growth of hydrothermal ZnO nanowires.

## Introduction

Hydrothermally grown ZnO nanowires have attracted significant attentions of many researchers in academia and industry due to their various functional properties, including optical and electrical properties^1–5^. The major useful feature of hydrothermal method is that whole hydrothermal processes can be performed under a relatively low-temperature range less than 100 °C^6,7^, which is hardly attainable to other conventional vapor-phase nanowire growth methods^8–13^. This low-temperature process expands the application range, particularly when integrating nanowires with other components on various substrates^2,4,14^. Preferential nucleation on ZnO (0001) polar plane is a key process for the anisotropic ZnO nanowire crystal growth^15–17^. Previous studies have revealed the roles of various parameters, such as pH, temperature, and ionic species on hydrothermal ZnO nanowire growth^18–27^. The origin of anisotropic crystal growth in hydrothermal ZnO nanowires has been interpreted in terms of the variations of ionic species in aqueous solutions and their electrostatic interactions with ZnO crystal planes^18,27^. Typically, these hydrothermal ZnO nanowires have been grown under an alkaline condition since divalent Zn ions (Zn^2+^) do not hydroxylate in acidic environments^25–30^. This is because zinc hydroxide complexes (Zn(OH)_n_) are necessary as precursors in solution for ZnO crystal growth^19,25^. A supplying rate of zinc hydroxide complexes to crystal growth interface on (0001) plane essentially determines a growth rate of hydrothermal ZnO nanowire growths^19,25^. Therefore, increasing a concentration of zinc hydroxide complexes in solution is effective to increase a growth rate of hydrothermal ZnO nanowires. However, there are inherent limitations for the enhancement of the nanowire growth rate within the framework of conventional strategy based on increasing a precursor concentration. First, the homogeneous nucleation in bulk solution occurs at the relatively low Zn concentration range^30^. This limits the supplying rate of Zn species to nanowire and the resultant growth rate of ZnO nanowires. To suppress this homogeneous nucleation in solution, an ammonia was added^27–30^. The resultant zinc ammonia complexes act as buffer for Zn species, which suppresses the homogeneous nucleation in solution even at relatively high Zn concentration range^27–30^. However, there is another inherent limitation of increasing Zn species concentration to keep an anisotropic crystal growth along [0001] direction. This is because a face selective anisotropic growth on (0001) plane emerges within a certain concentration range^16^. This is so-called as “concentration window”^16^. Above the concentration range, a crystal growth on $(10\overline{10})$ plane tends to simultaneously occur, which suppresses an anisotropic crystal growth on (0001) plane, promoting to be a film structure rather than a nanowire structure. The precise control of the Zn species concentration therefore is necessary to maintain the nanowire morphology without lateral $(10\overline{10})$ plane growth. This restriction of appropriate concentration range “concentration window” for anisotropic ZnO nanowire growths is essential. Although understanding what exactly determines the width of “concentration window” is important to design the anisotropic crystal growth process of ZnO nanowires, the fundamental knowledge as to “concentration window” is still scarce. It is not well understood how a growth condition affects the width of “concentration window”, for example the effect of ammonia addition, which is frequently employed to suppress a seed nucleation in solution^27–30^. These backgrounds motivated us to study the effect of ammonia addition on the width of “concentration window”, which is an essential requirement for the anisotropic nanowire crystal growth.

## Results and Discussions

### Effect of ammonia addition on concentration window width

**Figure 1:**
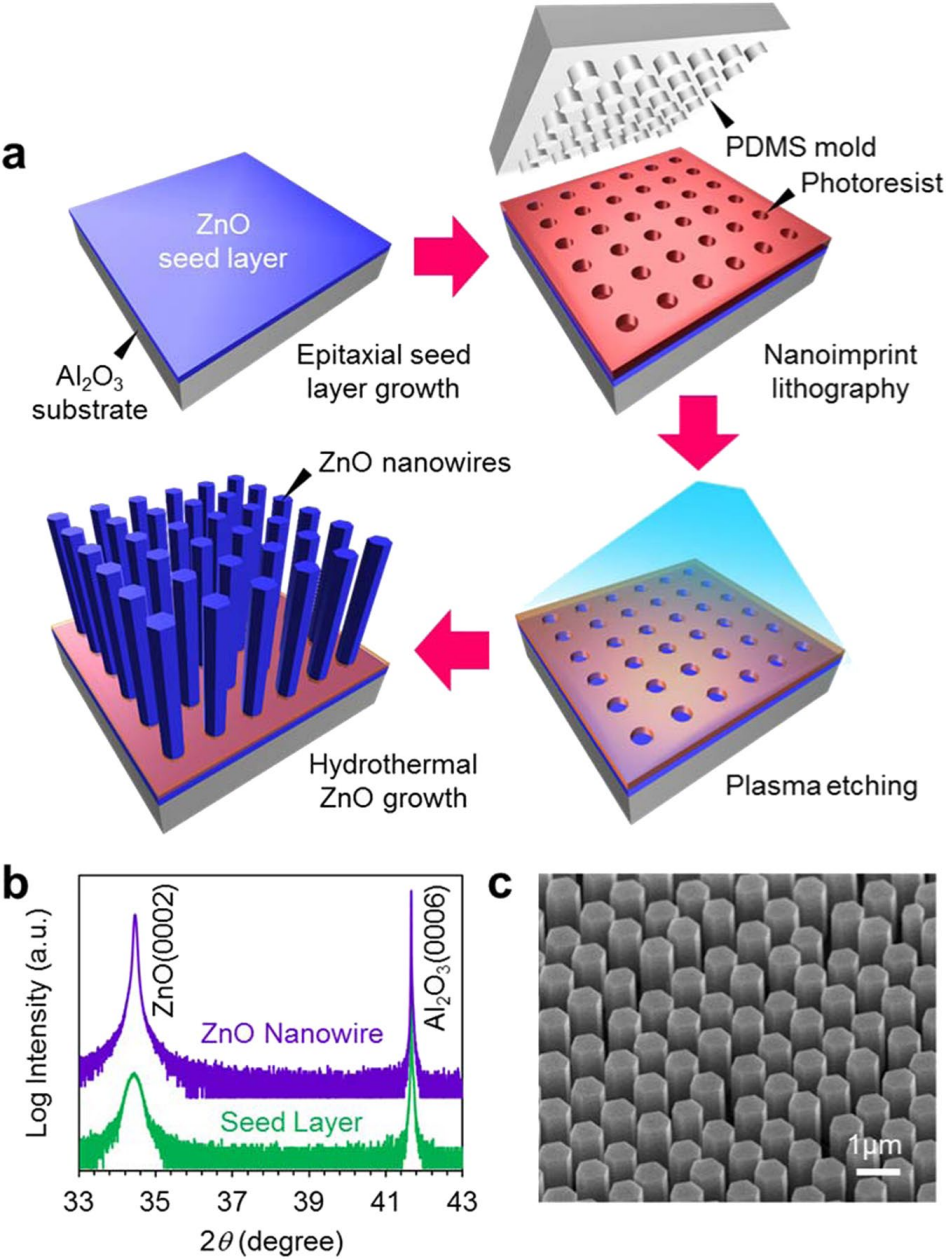
(**a**) Schematic of fabrication process for regular array of ZnO nanowires. (**b**) XRD data of ZnO seed layer and ZnO nanowires on Al_2_O_3_ substrate. (**c**) Typical SEM image of fabricated regular array of ZnO nanowires.

**Figure 2:**
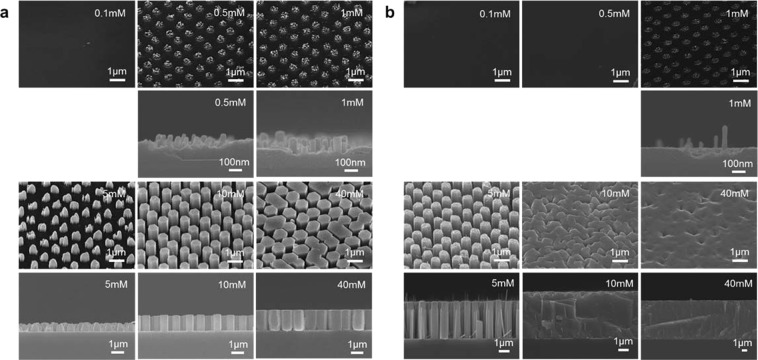
Effect of ammonia addition-500 mM on ZnO nanowire morphologies when varying concentration of zinc nitrate hexahydrate from 0.1 to 40 mM. All growth experiments are performed for 5 h. (**a**) SEM images of nanowires grown without ammonia, and (**b**) with excessive ammonia addition-500 mM. Upper shows tilted images and lower shows cross-sectional images, respectively.

**Figure 3:**
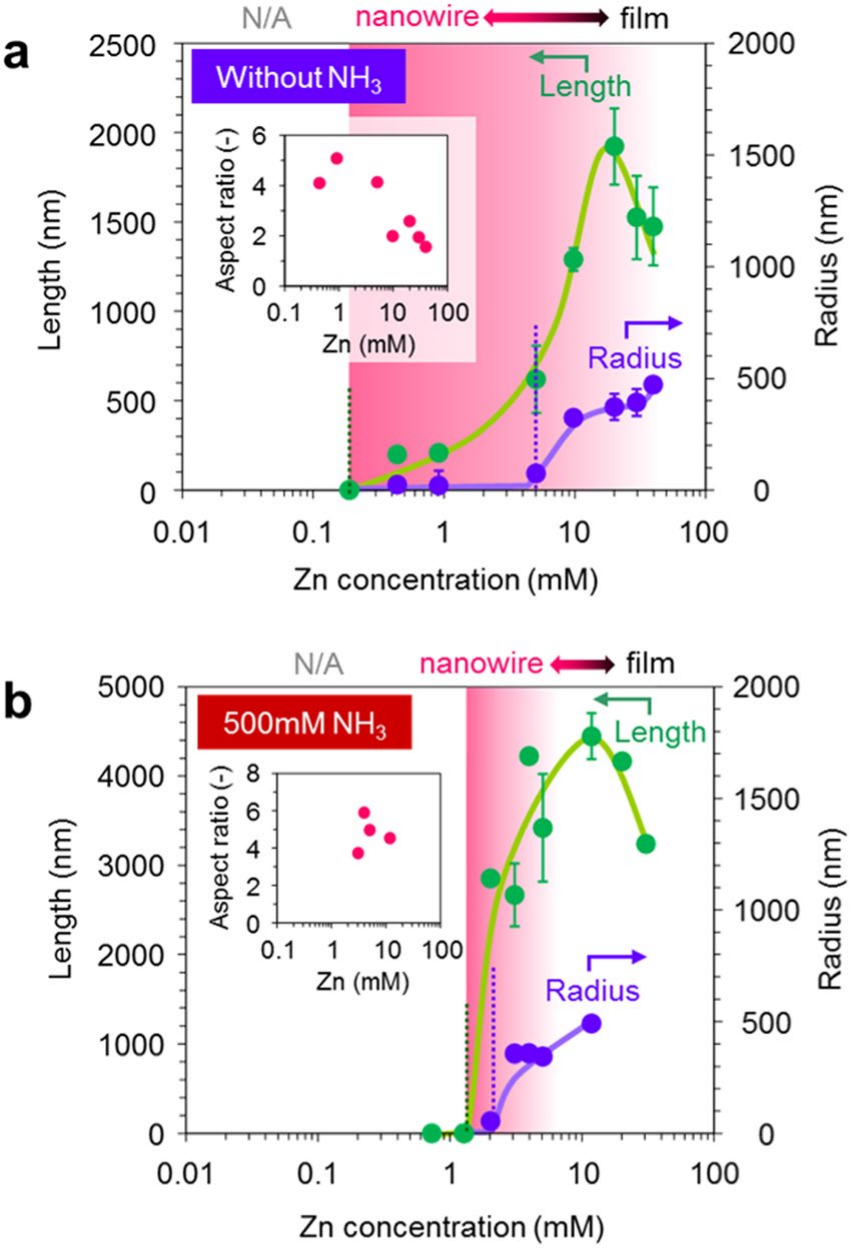
Zn concentration dependence on the nanowire morphology data (including length and radius), which are measured from SEM images of regular arrays. Inset shows aspect ratio. All growth experiments are performed for 5 h. (**a**) Zn concentration dependence on ZnO nanowire morphology with HMTA 15 mM without ammonia addition. (**b**) Zn concentration dependence on ZnO nanowire morphology with HMTA 15 mM with ammonia addition-500 mM. In both figures, the Zn concentration range, where a nanowire can be grown, is highlighted by a red color.

Figure 1a shows the fabrication process employed in this study. Epitaxial ZnO seed layers (33 nm thickness) are deposited onto single crystalline Al_2_O_3_ (0001) substrate by pulsed laser deposition^31^. The deposition temperature and the oxygen partial pressure during film deposition are 600 °C and 1 Pa, respectively. As shown in Fig. 1b, fabricated ZnO seed layers are grown along [0001] orientation. The regular arrays of ZnO nanowires grown along [0001] orientation are obtained as shown in Fig. 1b,c. Figure 2 shows the effect of ammonia addition-500 mM on the nanowire morphologies when varying the concentration of zinc nitrate hexahydrate in solution. The other experimental conditions are described in experimental method section. As seen in the SEM images, the appropriate concentration range “concentration window” for nanowire growth is consistently observed. Below the “concentration window”, there is no visible crystal growth. Within the “concentration window”, the nanowires tend to grow, and further increasing the concentration enhances a crystal growth even along lateral direction, which increases the nanowire diameter and finally showing a film structure rather than a nanowire structure. It should be highlighted that the ammonia addition substantially narrows the width of “concentration window”. Regarding the effect of ammonia addition, there are three remarkable differences on the concentration dependence data. First, the ammonia addition increases the critical concentration for nanowire growths. Second, the ammonia addition lowers the critical concentration for film structures. Third, the ammonia addition increases the nanowire growth rate. To specify more quantitatively above trends regarding the concentration dependence, we extract the length and radius data of fabricated nanowires as a function of a concentration, and the results are shown in Fig. 3. Data of nanowires grown for 5 h are shown in the figure. The length data and radius data are measured over 100 nanowires in cross-sectional SEM images or top-view SEM images. Above three major trends as to the effect of ammonia addition can be more quantitatively confirmed in Fig. 3. First, the ammonia addition shifts the critical concentrations for nanowire growth to the higher value-1.5 mM from 0.15 mM. Note that the critical concentration value (0.15 mM) for nanowire growth without the ammonia addition differs from the value (0.01–0.1 mM) of our early work^16^, this is because our early work employed an equimolar ratio of the two, whereas the present work used the constant concentration (15 mM) of hexamethylenetetramine (HMTA) when varying the concentration of zinc nitrate hexahydrate from 0.1 to 40 mM. On the other hand, the ammonia addition lowers the critical concentrations for lateral crystal growth from 5 mM to 2 mM. In addition, as to the nanowire growth rate at the critical concentration for lateral crystal growth, the nanowire growth rate with ammonia addition is 570 nm/h, which is almost 4 times higher than the nanowire growth rate (140 nm/h) without ammonia addition. These concentration data highlight that the ammonia addition narrows the width of “concentration window” with the increased nanowire growth rate.

### Discussion on concentration window width with ammonia addition

**Figure 4:**
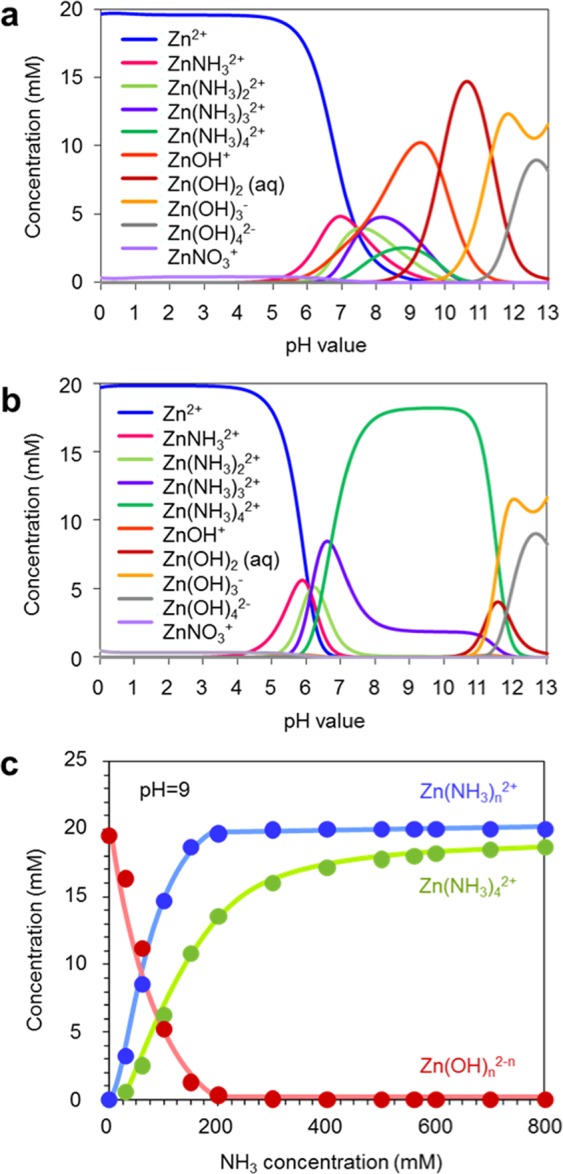
(**a**) Simulation data of ionic species in solution without ammonia addition (ammonia is supplied only by decomposition of HMTA). (**b**) Simulation data for solution with 500 mM ammonia addition. (**c**) Effect of ammonia addition on concentrations of various zinc complexes. In simulations, the temperature and Zn(NO_3_)_2_ concentration are set to be 95 °C and 20 mM, respectively.

We calculate the populations of existing ionic species in aqueous solution, as shown in Fig. 4. The thermodynamic calculations are performed using software-Visual MINTEQ. These calculation data reveal the effect of ammonia addition in terms of the population of ionic species in aqueous solution. Figure 4(a) shows the calculated population data of ionic species in solution without ammonia addition (ammonia is supplied only by decomposition of HMTA). Figure 4(b) shows the calculated population data of ionic species in solution with ammonia addition-500mM. In these figures, Zn^2+^, Zn(NH_4_)_*n*_, Zn(OH)_*x*_, ZnNO_3_^+^ are shown as the major ionic species in solution. When ammonia is not added, Zn^2+^ and zinc hydroxide complex ions coexist at pH values ranged from 6 to 10. The ammonia addition (500 mM) results in the increase of zinc ammonia complex ions^27–30^ via a ligand exchange process at pH values ranged from 6 to 11. These zinc ammonia complex ions tend to suppress the populations of zinc hydroxide complex ions at pH values ranged from 6 to 10, as increasing the ammonia concentration, as shown in Fig. 4c. The increase of zinc ammonia complexes lowers the degree of supersaturation of zinc hydroxide complex ions in the growth solution^27–30^, leading to the increase of critical concentration for nanowire growth as shown in Fig. 3b. Since a pH value strongly affects the populations of existing ionic species in aqueous solution as seen in Fig. 4, we measure pH values for growth conditions of experiments in Fig. 3. The pH values for experiments without ammonia addition are ranged from 6.5 to 8 when varying the concentration of zinc nitrate hexahydrate (0.1–40 mM), which is a weak acid. When ammonia-500 mM is added, the pH values are ranged from 10.2 to 11.3 for the concentration range (0.1–40 mM) of zinc nitrate hexahydrate. Thus, it is necessary to consider the effect of pH to understand the difference between Fig. 3a,b. Based on the pH value difference and thermodynamic calculation data in Fig. 4, we consider the effect of ammonia addition on the molar ratio of zinc hydroxide complex ions as precursors. The molar ratio of total zinc hydroxide complex ions in ammonia-500 mM added solution is estimated to be about 0.5 mM, which is approximately 5 times lower than the value-2.8 mM for solution without ammonia addition. Interestingly, the molar ratio difference of hydroxide complex ions is reasonably consistent with the critical concentration difference for nanowire growths in Fig. 3a,b (0.15–0.2 mM for case without ammonia and 1–1.5 mM for case with ammonia addition). This consistency implies that not zinc ammonia complex ions but hydroxide complex ions act as a precursor for ZnO nanowire growth. Thus, the increase of critical concentration for nanowire growth as shown in Fig. 3b can be interpreted in terms of the change of precursor complex concentration in solution. It is noted that in the presence of ammonia addition, the nanowire growth rate increases even when decreasing the total Zn concentration in solution.

Next we question why the critical concentration for lateral crystal growth of nanowires shifts to the lower value from 5 mM to 2 mM with the ammonia addition. This effect cannot be interpreted in terms of our above model based on reduced molar ratio of zinc hydroxide complex ions. Here we consider the difference of pH values due to the ammonia addition to explain this effect. An effective surface charge of crystal plane varies from a positive value to a negative value below and above an isoelectric point (IEP)^32,33^. It has been reported that IEP value for (0001) plane of ZnO is reported to be 8.7 ± 0.2, which is lower than that for $(10\overline{10})$ plane-10.2 ± 0.2^32,33^. In other words, the effective surface charge of $(10\overline{10})$ plane is always higher than that of (0001) plane at moderate pH range. At pH range 10–11 for ammonia addition experiments, both crystal planes must be charged more negatively than those at pH range 6.5–8 for experiments without ammonia addition. As seen in the populations of ionic species in aqueous solution, for ammonia addition experiments at pH range 10–11, the major positively charged ions are zinc ammonia complex ions-Zn(NH_3_)_4_^2+^, and the major negatively charged ions are NO_3_^−^. On the other hand, for experiments without ammonia addition at pH range 6.5–8, the major positively charged ions are -Zn^2+^, and the major negatively charged ions are NO_3_^−^. Since the crystal planes of ZnO at pH range-10–11 should be negatively charged, the major positively charged ions-Zn(NH_3_)_4_^2+^ should act as a counter ion, forming an electric double layer onto crystal planes. In this case, the rate limiting process for ZnO nanowire crystal growth is a ligand exchange reaction from zinc ammonia complexes surrounding the nanowire surface to hydroxide complexes. On the other hand, the major counter ions on positively charged crystal planes at pH range 6.5–8 are NO_3_^−^, which do not contain Zn. In this case, the diffusion of zinc hydroxide complex ions is the rate limiting process for ZnO nanowire crystal growth. Thus, these rather contrasting results as to a polarity infers that the critical concentration for lateral crystal growth of nanowires is reduced due to the accumulated positively charged Zn source ions at the crystal growth interface.

### Discussion on increased nanowire growth rate with ammonia addition

**Figure 5:**
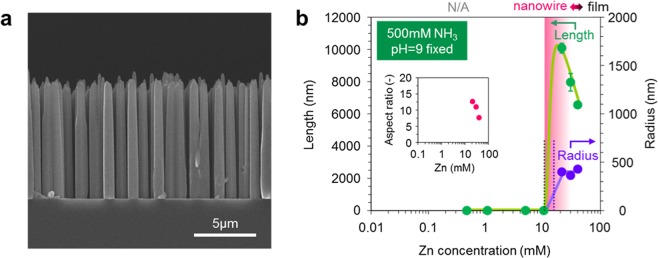
Effect of pH control on ZnO nanowire morphology data ((**a**) SEM image and (**b**) length and radius data) as a function of Zn concentration with HMTA 15 mM, excessive ammonia addition-500 mM and pH = 9 control. Inset of (**b**) shows aspect ratio. The Zn concentration range, where a nanowire can be grown, is highlighted by a red color.

Next we explain how the growth rate of ZnO nanowires can be enhanced when we control the zinc complex concentration within “concentration window”. The increased nanowire growth rate seems to be contradictive since a nanowire heterogeneous nucleation rate is reduced by a reduction of precursor complex (Zn(OH)_*x*_) concentrations. To explain this apparently contradictory observations of a reduced nanowire heterogeneous nucleation rate and an increased growth rate above the nucleation threshold, we consider the surface polarity variation and the distribution of zinc complex ion species. We propose a model based on a fast rate limiting process of a ligand exchange reaction between zinc ammonia complexes and hydroxide complexes (Zn(NH_3_)_*x*_ + H_2_O → Zn(OH)_*x*_ + NH_3_OH) from a diffusion dominant process. In the presence of excessive ammonia-500mM, the major zinc complexes are zinc ammonia complexes, as calculated in Fig. 4. Since zinc hydroxide complexes are a direct precursor for ZnO crystal growth, a ligand exchange reaction between zinc ammonia complexes and hydroxide complexes becomes a rate limiting process for the crystal growth. Considering the crystal growth situation near the interface, the zinc hydroxide complexes are surrounded by a plenty of zinc ammonia complexes. Since an equiblium zinc hydroxide complex concentration is substantially lower than that of zinc ammonia complexes, the consumed zinc hydroxide complex for crystal growth can be simultaneously compensated by a plenty of surrounding zinc ammonia complexes via the ligand exchange reaction. Considering the equiblium molar ratio between the two zinc complexes as calculated in Fig. 4, the reaction must be rather dynamic. Therefore, this fast ligand exchange reaction is important to understand the nanowire growth rate enhancement with ammonia addition. Based on this model, we further enhance a nanowire growth rate. Consider the thermodynamic calculation data (Fig. 4) as to the equilibrium molar ratio of zinc hydroxide complex ions when varying pH. As seen in Fig. 4b, the equilibrium molar ratio of zinc hydroxide complex ions can be further reduced at pH = 9 from pH = 10–11 for experiments in Fig. 3b. Therefore, we control the pH value by using HNO_3_. Figure 5 shows the Zn concentration dependence data of nanowire morphology when the pH value is set to be 9. As can be seen, the growth rate is drastically enhanced up to 2000 nm/h with maintaining the nanowire morphology, which is higher than 570 nm/h in Fig. 3b with ammonia addition and 140 nm/h in Fig. 3a without ammonia addition. As to the mechanism, looking at the critical concentration for nanowire growth is interesting, which is further increased to 10 mM. In fact, this value is almost 10 times higher than that for experiments (with ammonia addition at pH = 10–11) in Fig. 3b and 100 times higher than that for experiments (without ammonia at pH = 6.5–8) in Fig. 3a. These results highlight that controlling zinc complex concentration with pH control can further enhance the nanowire growth rate via further reducing the equilibrium molar ratio of zinc hydroxide complex ions. The pH control further narrows the width of “concentration window”.

### Effect of ammonia addition on physical properties of nanowires

**Figure 6:**
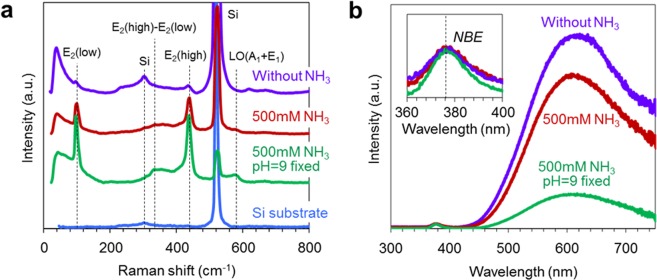
Effects of ammonia addition and pH control on physical properties of ZnO nanowires. (**a**) Raman spectra data and (**b**) PL spectra data.

**Figure 7:**
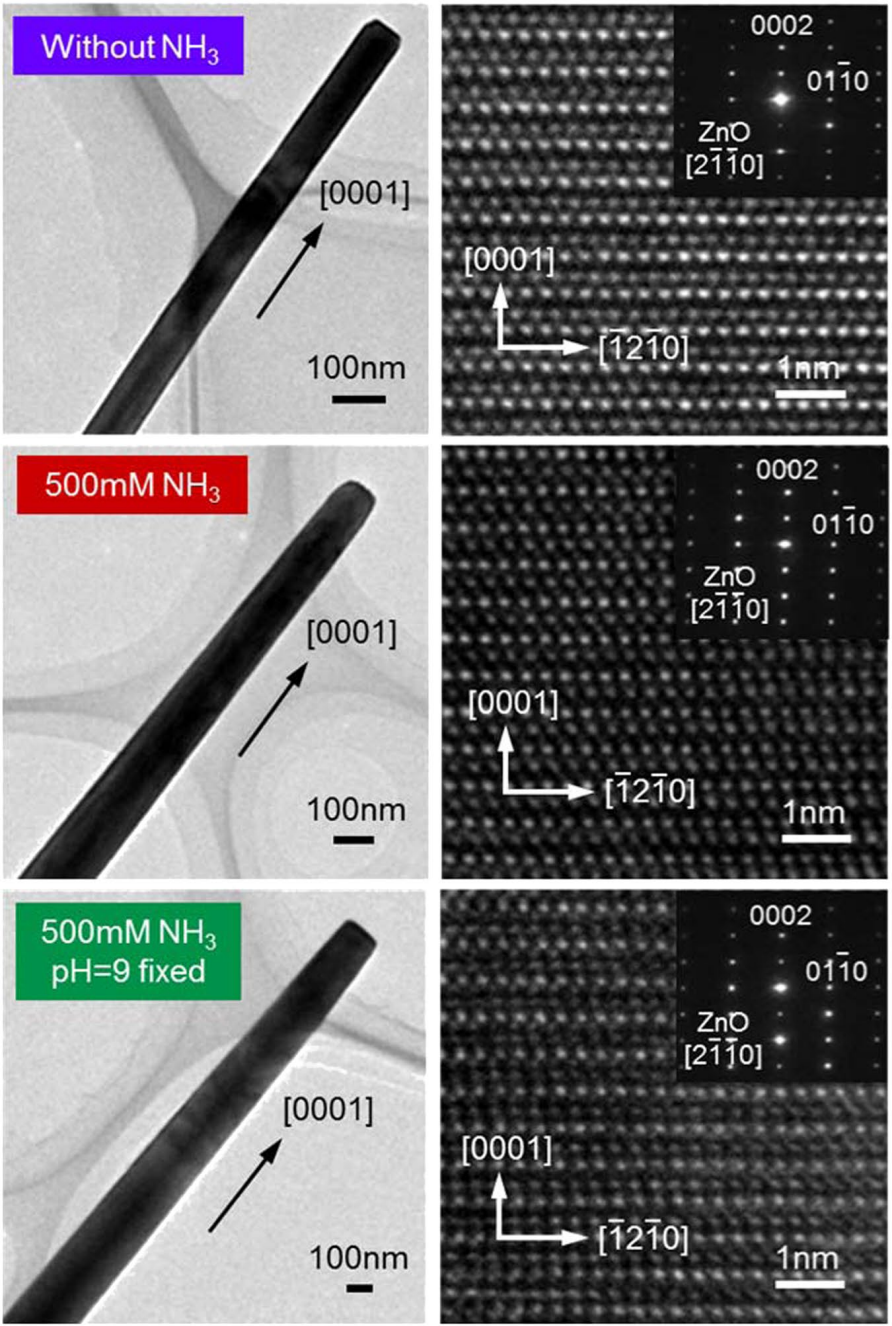
Effects of ammonia addition and pH control on TEM images of ZnO nanowires.

**Figure 8:**
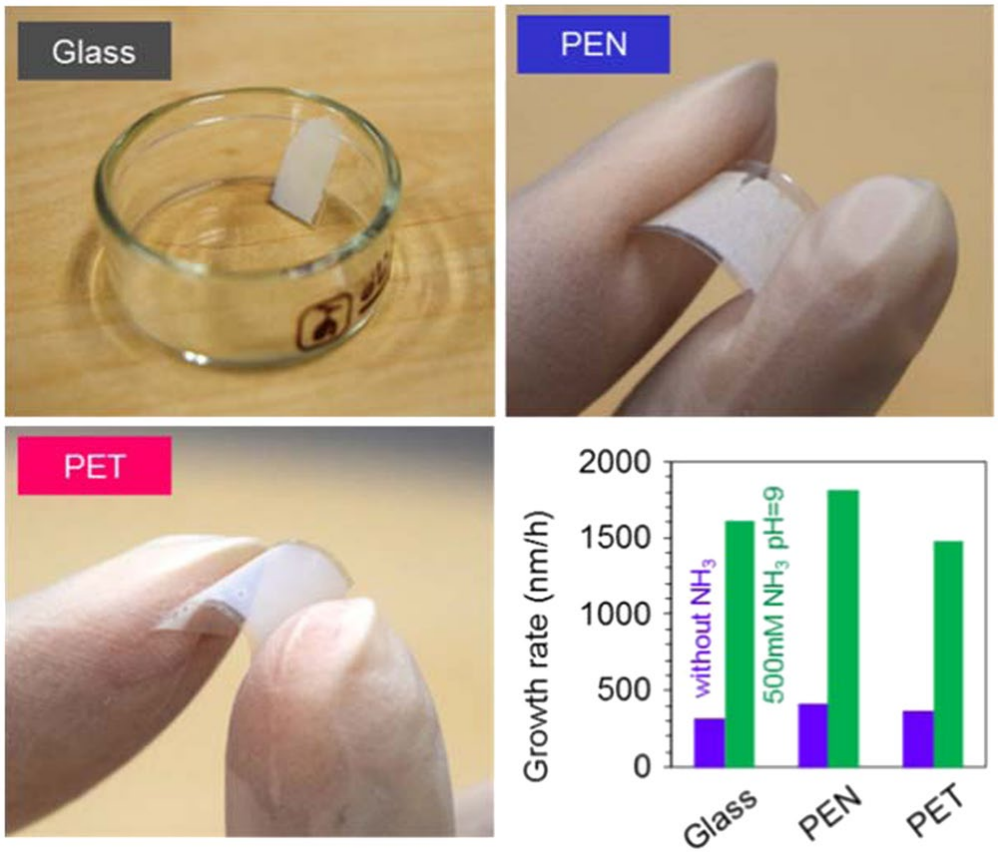
Applicability of the present strategy for hydrothermal ZnO nanowire growth for various substrates including glass, PEN and PET substrates (growth time: 5 h, growth temperature: 95 °C, and Zn concentration: 20 mM). For these experiments, the ZnO seed layers with polycrystalline form are deposited by sputtering at room temperature.

Finally, we examine the effect of ammonia addition on the physical properties of fabricated ZnO nanowires. Figure 6 show the Raman spectra data and the photoluminescence (PL) spectra data of fabricated ZnO nanowires. All measurements are performed at room temperature. For Raman spectroscopy measurement, we utilize a laser with excitation wavelength of 532 nm. The observed peaks are assigned as E_2_(low), E_2_(high), A_1_(TO), A_1_(LO), LO(A_1_ + E_1_), E_2_(high)-E_2_(low) phonon modes, which are prominent peaks of wurtzite ZnO structure^34–36^. We utilize E_2_(high) peak to evaluate the crystalline quality of ZnO nanowires, which is attributed to the vibration of oxygen sublattice^34,35^. The full width half maximum (FWHM) of high frequency E_2_ mode (E_2_(high)) peaks tends to decrease for ammonia addition and pH control, as shown in Table 1, highlighting the improvement of crystallinity. For the PL spectra data of fabricated ZnO nanowires, the broad peaks are commonly observed at the wavelength range of 500–750 nm, which are related to the deep level defects of ZnO. The intensity of spectra is normalized at near band edge emission (NBE) peaks as shown in inset. We find that the intensity of defect peaks tends to decrease by ammonia addition and pH control while the wavelength of both the NBE peaks and the defect peaks are almost unchanged, indicating the improvement of crystallinity as consistent with the Raman spectra data. Figure 7 shows the TEM images of fabricated ZnO nanowires. As can be seen, all ZnO nanowires consistently showed the single crystallinity. The TEM images show a tapering on both ammonia (and HNO_3_) addition samples. This is due to the effect of narrowed concentration window width, since the occurrence of film growth at nanowire sidewall tends to be not negligible in such narrowed concentration window. All data consistently demonstrate that the ammonia addition and pH control increase the crystallinity of ZnO nanowires. These trends are contradictive to the previous report of which the crystallinity of hydrothermal ZnO nanowires decreases by the ammonia addition^37^. One possible reason to explain these contradictive results is the difference of lateral growth. Since Watanabe *et al*. reported that the crystal grown on $(10\overline{10})$ plane contains more crystal imperfections than that grown on (0001) plane^38^, the decrease of crystallinity in previous study is presumably due to the promotion of crystal growth on $(10\overline{10})$ plane by ammonia addition. On the other hand, in this study, we suppress the lateral growth by precisely control the Zn concentration within the “concentration window”. Although further systematic investigations are needed to elucidate the exact roles ammonia and pH on the crystallinity of hydrothermal ZnO nanowires, these results indicate that “concentration window” principle allows us not only to enhance the anisotropic crystal growth but also to elucidate the intrinsic property of hydrothermal ZnO nanowires. All data consistently demonstrate that the ammonia addition does not significantly alter the physical properties of ZnO nanowires. In addition, we examine the applicability of the present method for various substrates including glass, flexible polyethylene naphthalate (PEN) and polyethylene terephthalate (PET) substrates, as shown in Fig. 8. Since the applicability of nanowire arrays on flexible substrates has been demonstrated^39^, the present methodology would further promote such applications of nanowires.

**Table 1:** FWHM of E_2_(high) peaks for Raman spectroscopy.

Without NH3	500 mM NH3	500 mM NH3 pH = 9 fixed
**FWHM of E2(high) peak (cm** ^**−1**^ **)**		
16.7	15.7	14.8

## Conclusion

In summary, we show the effect of ammonia addition on the width of “concentration window” using conventional hydrothermal ZnO nanowire growth. We found that the addition of ammonia substantially narrows the width of “concentration window”. Within the narrow range of zinc complex concentration, we found a significant enhancement of growth rate (up to 2000 nm/h) of ZnO nanowires. This concept of narrowed “concentration window” would be useful especially when it is necessary to increase a growth rate of hydrothermal ZnO nanowires without any additional equipment. The narrowed “concentration window” and the resultant growth enhancement by an ammonia addition can be understood in terms of two synchronized effects of both a reduction of zinc hydroxide complex concentration and a fast rate limiting process of ligand exchange between different zinc complexes when the critical nucleation emerges.

## Method

ZnO nanowire regular arrays are employed to measure a growth rate of each crystal plane. The ZnO nanowire regular arrays are fabricated onto spatially patterned photoresist/ZnO seed layer/Al_2_O_3_ substrates by utilizing hydrothermal method. Epitaxial ZnO seed layers (33 nm thickness) are deposited onto single crystalline Al_2_O_3_ (0001) substrate by pulsed laser deposition^31^. The deposition temperature and the oxygen partial pressure during film deposition are 600 °C and 1 Pa, respectively. Fabricated ZnO seed layers are grown along [0001] orientation. Photoresist-AZ5206/Z5200 (2:1) (Electronic Materials), which is utilized for a mask of nanowire regular array, is coated onto ZnO/Al_2_O_3_ substrate by spin-coating at 5000 rpm for 60 s and subsequently bake it at 120 °C for 2 min. After this process, the circular hole array patterns with 520 nm diameter and 400 nm interval distances are fabricated by nanoimprint lithography. The patterned photoresist is then solidified at 120 °C for 5 min. The photoresist residuals at the bottom of patterned hole are removed by reactive ion etching process (JEOL, JP-170). The remained photoresist thickness on nonpatterned area is ∼50 nm. Then, we perform the hydrothermal ZnO nanowire growth experiments. Solutions for hydrothermal reactions are mixtures composed of zinc nitrate hexahydrate-Zn(NO_3_)_2_•6H_2_O (Wako, 99.0%) and hexamethylenetetramine-HMTA, (CH_2_)_6_N_4_ (Wako, 99.0%) 15 mM. The aqueous solution is prepared at room temperature, and the zinc nitrate hexahydrate concentration is varied from 0.1 to 40 mM to examine the concentration dependence. For the ammonia contained growth condition, 500 mM ammonia aqueous solution is added after mixing zinc nitrate hexahydrate and HMTA. The pH values are measured by using a pH meter (EUTECH, Cyber Scan pH310). For the pH control condition, HNO_3_ is carefully added to the ammonia contained growth solution by monitoring the pH value. The patterned substrate is immersed into the solution and kept at 95 °C for a given time. Finally the regular arrays of ZnO nanowires grown along [0001] orientation are obtained. Structural characterizations of fabricated ZnO nanowires are performed by using x-ray diffraction (PHILIPS, X’Pert MRD 45 kV, 40 mA), field emission scanning electron microscopy-SEM (JEOL, JSM-7610F) and transmission electron microscopy-TEM (JEOL, JEM-ARM200F). Photoluminescence (JASCO, FP-8500), Raman spectroscopy (Tokyo Instruments, Nanofinder^®^30) and UV-vis absorption spectra (JASCO, V-770) are measured to examine the properties of nanowires. Visual MINTEQ software is employed to calculate equilibrium concentrations of various ionic species in solution at given temperature and pH ranges.
